# An Interview with Cesare Montecucco

**DOI:** 10.3390/toxins10080307

**Published:** 2018-07-24

**Authors:** Mandë Holford, Andreas Rummel

**Affiliations:** 1 Department of Chemistry and Biochemistry, Hunter College Belfer Research Building and CUNY Graduate Center, BRB 424, New York, NY 10021, USA; mholford@hunter.cuny.edu; 2 Institut für Toxikologie, Medizinische Hochschule Hannover, Carl-Neuberg-Str. 1, 30625 Hannover, Germany; Rummel.Andreas@mh-hannover.de

## Abstract

Cesare Montecucco is a member of the board of various leading scientific journals. His research focus is on the regeneration of the peripheral nervous system and on the mechanisms of action of toxins and related diseases, including tetanus, botulism, anthrax and Helicobacter pylori-associated diseases. In this interview, we talked to Cesare Montecucco about his career, learning and culture.

Cesare Montecucco is an Italian pathologist and full professor at University of Padova, Italy. He is a member of the European Molecular Biology Organization, the German Academy of Sciences Leopoldina, the Italian Accademia dei Lincei, the Istituto Veneto di Scienze Lettere ed Arti, the Academia Europaea and the European and American Academies of Microbiology. He has received several scientific prizes including the Harvard Medical School Shipley Prize for Medicine in 1993, the Feltrinelli Prize for Medicine in 2004, the Redi Award in 2009 and the Ehrlich Prize for Medicine in 2011 (Figure 1). In addition to Padova, he has performed research at the University of Cambridge, Institut Pasteur in Paris, EMBL, Universities of Utrecht and Costa Rica in San Josè.

## 1. Career

**Q. How was that you started to work on toxins?**

At the end of my first post-doc in Cambridge (UK) where I worked on lipid-protein interaction with Jim Metcalfe and then on the measurement of membrane potential in small cells using liposoluble fluorescent membrane probes with the Roger Tsien, the 2008 Nobel Prize laureate for chemistry, also for family reasons, I decided to go back to my alma mater, the University of Padova where I had graduated in chemistry and then in biology. My initial mentor Prof. Azzi had letft for Berne and Prof. Azzone offered me another post-doctoral position to work on mitochondria, but I contracted to spend 30% of my time on a subject of my choice.

I was driven by seminars and readings done in Cambridge on developments of the theory of biological evolution, including papers of Leigh Van Valen and of William Hamilton, who introduced the concepts of the “Red Queen” and of the “Arm Race” suggesting the fundamental role played by parasites in biological evolution pattern and processes, from antagonistic coevolution to the origin of sex. Moreover, the reading of the fundamental books of Claude Bernard “Introduction à l’etude de la médecine expérimentale” and “Leçons sur les propriétés des tissus vivants”, in which he described his work on the action of curare, was very inspiring from a methodological point of view. These inputs led me to conclude that a privileged point of observation of nature for me would have been the host-pathogen interface, where co-evolution shapes virulence factors and defence mechanisms. Thus, I wrote a lab manifesto, which is still valid nowadays and can be summarized as follows. The study of the mechanism of action of animal and bacterial toxins, and of virulence factors in general, is important because it may lead to: (1) understanding of the pathogenesis of the disease caused by the pathogen; (2) learning about specific physiological functions of the host; (3) development of novel therapeutics (vaccines, inhibitors, etc.) against the disease under study; (4) the use of the virulence factor itself as a therapeutic (this latter point was derived later from the study of the literature on botulinum neurotoxins).

**Q. How did you begun to study clostridial neurotoxins?**

In 1984 I was spending a semi-sabbatical at the Institut Pasteur in Paris in the laboratory of Jean-Pierre Changeux, a great Renaissance-type of scientist who played a major role in the development of the concept of allostery and in the Monod-Wyman-Changeux model which correctly explains this fundamental property of biochemistry and cell biology. JPC had used the snake toxin α-bungarotoxin, isolated and studied by Chang and Lee in Taiwan, to affinity purify the acetyl choline receptor and to prove that this receptor is an allosteric transmembrane ion channel. I was working in his lab on the 5th floor of Batiment Monod and sleeping on the 7th floor, working extended schedules dictated only by the experiments, but I also had long hours to read. Jean-Pierre had a personal library that included several biochemistry and neurobiology journals, available to lab members. It was there that I read and studied in details about tetanus and botulinum neurotoxins. Luckily, on the other side of Rue du Dr. Roux, Patrice Boquet and Michelle Roa were studying the mechanism of action of tetanus toxin. I went to talk to them and we started to collaborate. After further specific readings and the development of the double receptor model for the neurospecific binding of these neurotoxins (TIBS 1986) I started a series of studies which is still going on after 34 years…… though the currently major line of research of the lab is the study of the mechanisms and molecules involved in regeneration of peripheral synapses and neurons after injury.

**Q. Throughout your scientific career in studying neurotoxins and their mechanism of action, what has led to the most helpful advancement in neurophysiology technology to help facilitate your understanding of neurophysiology? Can you clarify and simplify this question?**

During my two experimental master thesis and first post-doctoral work I had developed a competence on protein chemistry and membrane biology. During the work in Cambridge with Tim Rink and Roger Tsien I learned about platelets, red blood cells and lymphocytes, but I had no neurobiology culture. That I developed in Changeux’s lab, then on our own in Padova together with Giampietro Schiavo and during collaborations with Bernard Poulain then in Gif-sur-Yvette, with Fabio Benfenati then in Modena, with Stefan Catsicas then at Serono in Geneva, and later on with Michela Matteoli and Flavia Valtorta in Milan.

**Q. How has your research challenged previous knowledge about neurophysiology?**

I would say that we have added understanding and molecular notions that have discriminated among different hypotheses and possibilities more than we have ourselves challenged beliefs in neurophysiology. But we did challenge neuropathology, because in 1992 we disproved that tetanus and botulism are characterized by opposite symptoms, i.e., a spastic versus a flaccid neuroparalysis, because tetanus and botulinum neurotoxins have different mechanisms of action. Indeed, we proved that the two diseases are caused by the same molecular action, i.e., the cleavage of a single protein essential for the release of neurotransmitter. The opposite symptoms are simply due to the different neurons targeted by tetanus and botulinum neurotoxins: the inhibitory interneurons of the spinal cord and the peripheral cholinergic neurons, respectively. This was a real breakthrough in the understanding of the molecular pathogenesis of these diseases.

**Q. What has been your biggest obstacle in studying bacterial neurotoxins and their effects on neurophysiology?**

The major obstacle was trivial and unsurmountable at the same time: in the Department of Biomedical Sciences we were, and still are, not allowed to grow pathogenic Clostridia, whilst we can handle very limited amounts of toxins and cultivate other bacteria such as the human only pathogen Helicobacter pylori, which was the center of my attention for over twelve years. This was a real hurdle in the attempt to crystallize tetanus neurotoxins made by Giampietro Schiavo and myself, an effort that went on for many years (1986–1992), because we were convinced that structure would have told us its biochemical activity. This involved the purification of dozens and dozens of milligrams of a very toxic protein from sterile culture filtrates that were provided by Rino Rappuoli of the Italian vaccine company Sclavo. We had no specific permit, but we did it as we felt safe because everybody in the Institute was vaccinated against tetanus and because we were handling the large quantities of toxin during weekends and vacations when everybody else was away.

**Q. What do you think has been your greatest finding, or advancement, in your research of neurotoxins and neurophysiology?**

Our major findings concerning the mode of action of tetanus and botulinum neurotoxins can be summarized as follows.

On bacterial neurotoxins: the experimental demonstration, reported in 1992, that the N-terminal 50 kDa domains (L chain) of tetanus and botulinum neurotoxins are metalloproteases. This achievement was obtained using a variety of biochemical techniques and identified a novel family of metalloproteases, now dubbed M27, whilst the very similar active site metalloprotease anthrax lethal factor, whose substrate specificity we co-discovered in 1998, makes the metalloprotease family M34.

On bacterial neurotoxins and neurophysiology: (1) the demonstration that the L chains of these neurotoxins act in the cytosol of neurons by single site proteolysis of very selected substrates, i.e., the three proteins dubbed by Jim Rothman SNARE proteins (1992–1994), which are: VAMP, an integral membrane protein of synaptic vesicles, SNAP-25 and syntaxin, located mainly on the cytosolic side of the plasma membrane; (2) the identification of their sites of cleavage by the then 8 known clostridial neurotoxins. Important contributions were provided to these two points also by the groups of Reinhard Jahn in Yale and of Heiner Niemann in Giessen; (3) the proposal and initial experimental evidence that the molecular basis of such exceptional specificity is based on a double recognition of the substrate, i.e., of the cleavage site region and of other regions outside the cleavage region that we then termed SNARE motif. We now know, thanks to the work of Brunger’s, Rummel’s and of Swaminatham’s lab and others, that additional regions are involved; (4) we also substantially contributed to the knowledge that the neurotoxins are internalized via synaptic vesicle endocytosis; (5) more recently we demonstrated that an essential step of the entry of the L chain in the neuronal cytosol is the reduction of the interchain disulfide bond linking the L and H chains by a synaptic vesicle thioredoxin reductase-thioredoxin redox system. So much so that specific inhibitors of this redox system also prevents the neuroparalytic activity of the botulinum neurotoxins and the death by botulism.

Finding (1) was very important and impacted on the larger communities of neuroscientists and cell biologists. For several reasons. The fact that the single site proteolysis of VAMP is sufficient to cause neuroparalysis was important in neurophysiology because: (a) it proved beyond any possible doubt that the quantal release hypothesis of Bernard Katz was correct and (b) it identified the first synaptic vesicle molecule involved in the process of neurotransmitter release at the synapse. (c) It also provided a simple explanation for the fact there are animals largely resistant to tetanus such as rats and chickens; indeed we found that rats and chickens carry a mutation at the cleavage site of the VAMP isoform predominant in their spinal cord rendering it non cleavable by tetanus neurotoxin.

This reply is perhaps already too long, but let me recall here because of its relevance for molecular neurophysiology also the discovery (2004–2008) of the mechanism of action of the PLA2 snake neurotoxins that cause, like botulinum neurotoxins, a reversible peripheral neuroparalysis. The readings of clinical papers describing this reversibility in humans, similar to botulism, lead me to work with these presynaptic neurotoxins. In a rather short time period, we demonstrated that neurotoxicity was due to their PLA2 activity because cone-shaped fatty acids and inverted cone-shaped lysophospholipids are produced in the membrane. Such lipids favour synaptic vesicle fusion with the presynaptic membrane and inhibit their endocytosis. Lysophospholipids also form transient lipidic pores that mediate Ca^2+^ entry into the cytosol which causes the complete degeneration of nerve terminals. This was important for neurophysiology because it provided an important evidence for the fact that ready-to-release synaptic vesicles at nerve terminals are likely to be hemi-fused with the plasma membrane, a concept developed at the beginning of the 1980s by a group of talented and innovative Russian biophysicists in Moscow to explain membrane fusion.

This work was particularly important also for our lab because I was induced to propose, at the Redi Lecture in 2009 at the world IST Congress near Recife in Brazil, that the PLA2 snake neurotoxins and the spider alpha-latrotoxin could be used as tools for the understanding of the motor axon terminal degeneration and regeneration because their action is rapid (few hours), cause no inflammation, and it is reversible within few days in mice. Indeed, we then proved that these neurotoxins provide an ideal model to study peripheral neuroregeneration.

## 2. Learning

**Q. What advice(s) would you give to early career venom and protein toxin researchers?**

First, a scientific one. That of choosing an unsolved problem of general interest impacting on different aspects of biology and, possibly, medicine. Then I would suggest to: (i) pay visits to those scientists working on the chosen problem and (ii) attempt to use innovative experimental approaches. I would also advise a young person needing to use a complicated or expensive technical approach to collaborate with experts in the technique rather than spending years in building up his or her own knowledge. In simpler words choose your way and then try to walk as light and as fast as possible to your scientific destination.

I would also recommend to be curious not only about all aspects of the chosen problem but also of biology and biomedicine in general, by going through, say, ten multidisciplinary scientific journals, through the titles of major review journals and also by going to meetings that are not specifically covering your subjects. This will help in being original. 

A final, but strong, suggestion is that or reaching a complete awareness of the fact that a scientist indeed does a splendid job which is incomprehensible to the majority of the society, but, at the same time, this job gives you responsibility. That of being active in spreading the knowledge of science and of the scientific method and of practicing the ethics and the rigor of doing science. These are neglected aspects in today's society and it is the task of every scientist at all levels to spread these values.

**Q. What makes you optimistic about the future of venom and toxin research?**

The scientific richness of the field. There so many biological aspects of predator/prey or of host/pathogen interactions! And such a large variety of venom components and virulence factors! Not to forget the interactions and synergies among venom components and virulence factors that is almost unexplored. An example of this latter issue is the experimental evidence we provided together with Tatiana Baldari that the anthrax edema factor and the anthrax lethal factor cooperate actively to suppress the immune response.

**Q. What’s a belief you used to have about venom research that no longer holds up?**

In the initial period of my research, I preferred a reductionist approach, one that involved the purification of single components and to study their structure and molecular function. This approach is still valid, but we are now in the condition of beginning to make a synthesis of the single biological activities produced by toxigenic organism to understand the general picture of the predator/prey interaction. This is also important in order to understand the evolution and life style of the pathogenic organism.

**Q. Do you have a purpose or specific passion that you’re dedicated to?**

My general purpose which has often become a passion is that of fulfilling my curiosity which is largely, but not only, focused on science. In other words, is that learning as much as possible about a biological or a biomedical process and then trying to make my own opinion, or hypothesis about the underpinning mechanisms to be then tested experimentally within a reasonable amount of time. If it takes too long or it would require a too large investment of labour and money (rather frequent events in Italy) I get annoyed and bored and, sooner or later, I lose interest.

**Q. What has been the largest shift in your thinking or world view between now and your youth?**

There have been quite a few. Remaining in the field of biomedicine I have witnessed a decrease of the classical scientific approach of Galileo (he was at the University of Padova for 18 years during which he performed most of his classical experiments including the astrophysical ones). That is to say: observe a phenomenon, elaborate a hypothesis or a physical model and then design experiments to prove or disprove the hypothesis. In the positive case, perform additional experiments to assemble sufficient experimental evidence to promote the hypothesis to the state of a theory. First it comes an idea or a physical model and then the appropriate experiments. The proportion of discussions, time, and effort spent in the elaboration of ideas and hypothesis has progressively decreased in biology and biomedicine in favour of experiments and data collection. Indeed the Galilean procedure was prevalent after World War two until say 1965 with the α-helix model of proteins, the DNA double helix, the Hodgkin-Huxley theory of nerve conduction, the permease of Monod, the clonal selection theory, the chemiosmotic theory of Mitchell, the operon model and the allostery model of Monod, Jacob and Changeux. Nowadays, we are invaded by a flurry of phenomenology: observations over observations, all sort of screenings and data collections provide an immense body of information. But we badly need mechanistic understanding which is the only to way of providing a sense. In addition, we are more than often facing very complicated papers involving large number of KO mice, of genetically edited cells and animals, complicated protocols and elaborated imaging techniques. And too frequently conclusions are not supported by a significant number of repeated experiments with an appropriate statistics. The net result is that it may be difficult to repeat the experiments described in several biomedicine papers. I am becoming more and more disheartened and disenchanted and I am seriously worried about this state of facts. The more so as this situation is in part generated, and surely promoted, by an ever growing, but already gigantic, editorial business apparatus which brings with itself a general decrease of the scientific and critical levels of the reviewing process. In Darwinian terms this corresponds to the generation of too many variants with a concomitant decrease of the selection process. Darwinially worrying!

## 3. Culture

**Q. Is there a book you’ve read that you’d recommend universally (i.e., to everyone you meet)?**

This question makes me uncomfortable as there are so many “fundamental” books. Perhaps I would put number one Charles Darwin’s *The Descent of Man, and Selection in Relation to Sex*.

**Q. Are you on social media and what impact do you think it has/will have on the practicing of the scientific enterprise?**

No, I am afraid, not even on Linkedin which is very useful for work contacts. I doubt that more general social media are useful for scientists as valuable messages are drowned in an ocean of irrelevance or, worse, of an increasingly anti-scientific attitude. Part of my decision of staying out of social media is that I am afraid of the behaviour of the owners of social media and I am not ready to subscribe all their request of loss of privacy. Moreover, I have little time to spare, and it takes so much time to follow the social media where it is also so easy to get distracted! For the communication of my science I believe traditional media are better as they provide a filter and, as we know from Darwin, the selection process is as important as the generation of variations.

**Q. Where are your sources of information, where do you get your daily news about host-pathogen research?**

I am addicted to reading scientific papers and newspapers. I also perform almost daily literature searches using the common web facilities and databases. In addition, I continuously exchange information with colleagues at scientific meetings and via Skype or e-mails. I also try to go to as many seminars as possible in Padova, which is a University city that facilitates contacts.

**Q. If you were to create a venom superhero which organism would you use to depict as the character and what would be the superhero name?**

Difficult to choose a number one, again, but, perhaps I would put Escherichia coli because of its diffusion in the world and in laboratories and because of its immense capacity to variate by mutations and exchange of DNA. But if you are interested in knowing my preferred venomous animals, I am definitively for spiders. They make up a fascinating world in themselves.

**Q. Do you buy venomous animal paraphernalia, or have a museum like collection of any venomous animals, such as venomous insect collection?**

No, but I am very interested in the animals that I see in the house and in my mountain excursions. I am also a frequent visitor of natural science museums.

## Figures and Tables

**Figure 1:**
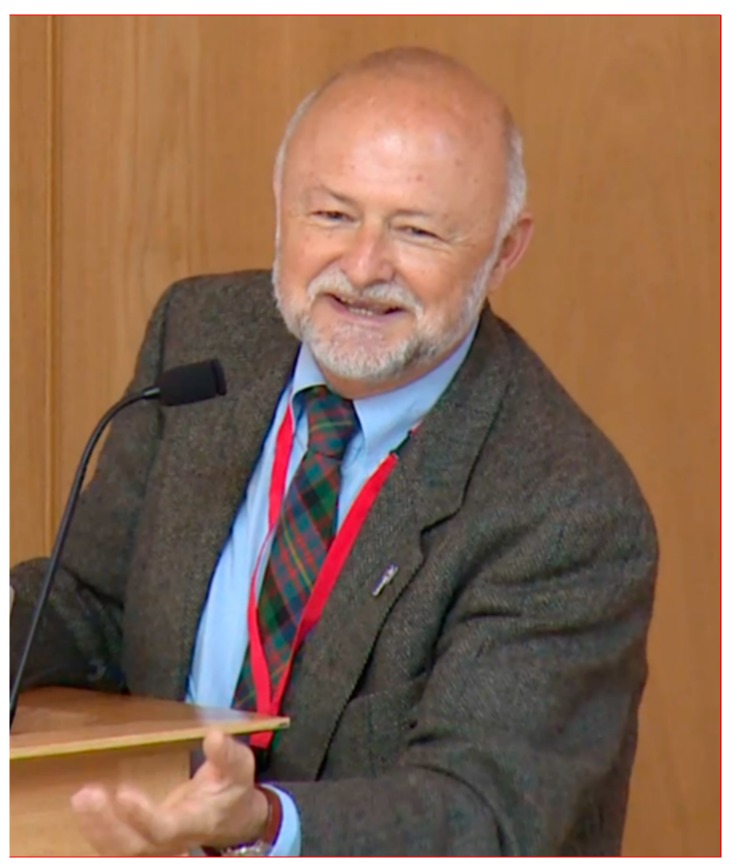
Cesare Montecucco.

